# Membrane Inlet Mass Spectrometry: A Powerful Tool for Algal Research

**DOI:** 10.3389/fpls.2020.01302

**Published:** 2020-09-04

**Authors:** Adrien Burlacot, François Burlacot, Yonghua Li-Beisson, Gilles Peltier

**Affiliations:** Aix Marseille Univ, Commissariat à l’énergie Atomique et aux énergies Alternatives (CEA), Centre National de la Recherche Scientifique (CNRS), Institut de Biosciences et Biotechnologies d'Aix- Marseille (BIAM), CEA Cadarache, Saint Paul-Lez-Durance, France

**Keywords:** gas exchange, photosynthesis, carbonic anhydrase, CO_2_ concentrating mechanism, O_2_ evolution, H_2_ production, microalgae, cyanobacteria

## Abstract

Since the first great oxygenation event, photosynthetic microorganisms have continuously shaped the Earth’s atmosphere. Studying biological mechanisms involved in the interaction between microalgae and cyanobacteria with the Earth’s atmosphere requires the monitoring of gas exchange. Membrane inlet mass spectrometry (MIMS) has been developed in the early 1960s to study gas exchange mechanisms of photosynthetic cells. It has since played an important role in investigating various cellular processes that involve gaseous compounds (O_2_, CO_2_, NO, or H_2_) and in characterizing enzymatic activities *in vitro* or *in vivo*. With the development of affordable mass spectrometers, MIMS is gaining wide popularity and is now used by an increasing number of laboratories. However, it still requires an important theory and practical considerations to be used. Here, we provide a practical guide describing the current technical basis of a MIMS setup and the general principles of data processing. We further review how MIMS can be used to study various aspects of algal research and discuss how MIMS will be useful in addressing future scientific challenges.

## Highlights

Photosynthetic microoganisms are major actors shaping the Earth’s atmosphere and limiting global warming by fixing CO_2_. We hereby describe practical and theoretical state of the art of the most polyvalent technique to measure gas exchange in microorganisms.

## Introduction

Since its formation, the Earth’s atmosphere has continuously been shaped by living organisms. Among all biological processes, oxygenic photosynthesis has dramatically changed the atmosphere composition by massively capturing CO_2_ and producing O_2_ during the Great Oxygenation Event that began 2.4 billion years ago with the emergence of cyanobacteria (Hohmann-Marriott and Blankenship, 2011). Nowadays, microalgae and cyanobacteria account for more than 50% of global photosynthesis (Field et al., 1998) and have a great importance in the regulation of atmospheric CO_2_ levels and global warming. Understanding biological mechanisms underlying CO_2_ capture or production of other greenhouse gases such as nitrous oxide (N_2_O) by microbial photosynthesis is of utmost relevance to better assess the impact of global changes on oceanic carbon sinks. It is also crucial to explore the limits of biomass productivity of algae and give some hints to assess the impact of microalgae-based biofuels on the environment (Burlacot et al., 2020a).

In the early 60s, Hoch and Kok (1963) designed a membrane inlet system coupled to a mass spectrometer (MIMS), which allowed the direct measurement of concentrations of dissolved CO_2_ and O_2_ in a microalgal suspension. In the MIMS setup they developed, a thin plastic membrane (polyethylene or Teflon) permeable to gases allowed part of the dissolved gases to pass from the microalgal suspension to the mass spectrometer. Coupled to the use of ^18^O-labeled O_2_, MIMS allowed *in situ* measurement of O_2_ uptake processes occurring during microalgal and cyanobacterial photosynthesis (Hoch and Kok, 1963; Gerster et al., 1974; Radmer and Kok, 1976; Gerster et al., 1977; Radmer and Ollinger, 1980b; Peltier and Thibault, 1985a; Peltier and Thibault, 1985b). The use of isotope-labeled water has also been an important tool for the study of water oxidation mechanisms by photosystem II (PSII) (Radmer and Ollinger, 1980a; Radmer and Ollinger, 1986; Shevela et al., 2011; Koroidov et al., 2014), which is not reviewed here and readers are referred to a recent review (Shevela and Messinger, 2013).

Later on, MIMS has been used to monitor various biological gases in the context of: *i*, inorganic carbon transport (Badger et al., 1994; Yu et al., 1994; Sültemeyer et al., 1998); *ii*, hydrogen production (van der Oost and Cox, 1988; Redding et al., 1999; Cournac et al., 2002; Tamburic et al., 2011); or *iii*, ethylene production (Zavřel et al., 2016). With the development of genetics tools, MIMS has been widely used to characterize various microalgae or cyanobacteria mutants and contributed to the understanding of molecular mechanisms involved in photosynthetic gas exchange (So et al., 1998; Hanson et al., 2003; Helman et al., 2003; Cournac et al., 2004; Jans et al., 2008). It has also been used for *in vitro* studies of enzymes using gases as a substrate or products, like hydrogenases (Leroux et al., 2008), carbonic anhydrases (Northrop and Simpson, 1998) and the Fatty Acid Photodecarboxylase (Sorigué et al., 2017). In addition, it has also been used to study artificial catalysts for water oxidation (Poulsen et al., 2005; Koroidov et al., 2015) and to follow biogeochemical cycles in the oceans (Chua et al., 2016). Taken together, MIMS has made significant contributions to the fields of photosynthesis research, enzymology, biofuel research, and earth science which was recently reviewed (Burlacot et al., 2020a).

MIMS is nowadays a mature technique implicated in a growing number of applications in the field of algal research. With mass spectrometers being more affordable in the past years, MIMS is increasingly available in many laboratories. However, mounting and running a MIMS setup is technically and theoretically challenging. Previous reviews have focused on some specific aspects of MIMS in the context of photosynthesis research (Degn, 1992; Kotiaho and Lauritsen, 2002; Konermann et al., 2008; Beckmann et al., 2009; Shevela and Messinger, 2013; Cheah et al., 2014; Shevela et al., 2018). The current review intends to provide a guide covering theoretical, technical, and data processing aspects required for a broad usage of MIMS in algal research.

## MIMS Setup, Optimization and Data Processing

**Figure 1:**
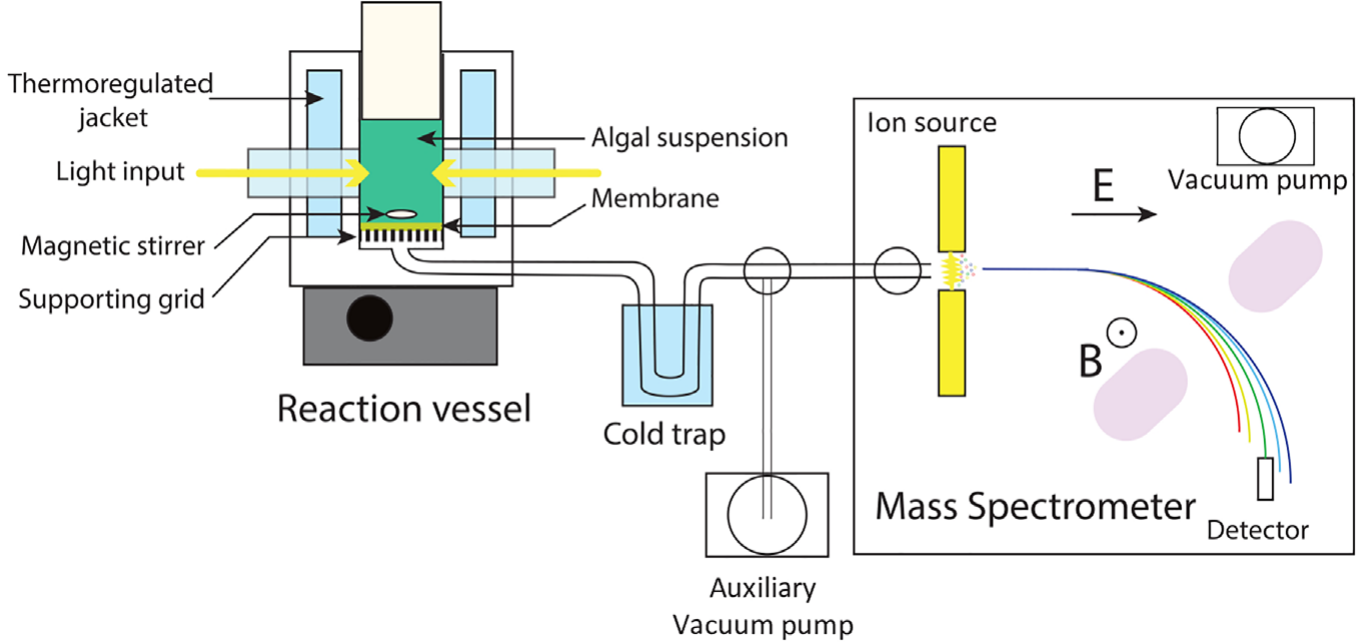
Schematic setup of a Membrane Inlet Mass Spectrometry. The biological sample (cell suspension, cell free extract, or enzyme containing medium) is introduced inside a thermo-regulated reaction vessel equipped with a gas permeable membrane. Dissolved gases contained in the liquid medium pass through the membrane to a high vacuum tubing. After passing through a cold trap protecting the mass spectrometer from water leaks, gases enter the ion source of a mass spectrometer. Upon ionization, charged molecules are accelerated by an electric field (E), deviated by a magnetic field (B), and collected by a detector. An auxiliary vacuum pump allows for making the vacuum on the line with the closed inlet to the mass spectrometer. B, magnetic field; E, electric field.

A typical MIMS setup is composed of five parts (**Figure 1**): (i), a reaction vessel containing the liquid medium/algal culture where gas exchange reactions take place; (ii), a membrane separating the liquid phase of the reaction vessel from the high vacuum line; (iii), a vacuum line connecting the reaction vessel to the mass spectrometer; (iv), a cold trap protecting the mass spectrometer from water leakage; and (v), a mass spectrometer for gas analysis. MIMS allows measurement of any volatile compound, with a molar mass up to a hundred gram per mole, dissolved in liquid phase (Kotiaho and Lauritsen, 2002). MIMS operation requires proper material setup and appropriate data processing. Each of the technical parts needs to be optimized to ensure reliable and stable measurement.

### The Membrane Inlet

**Figure 2:**
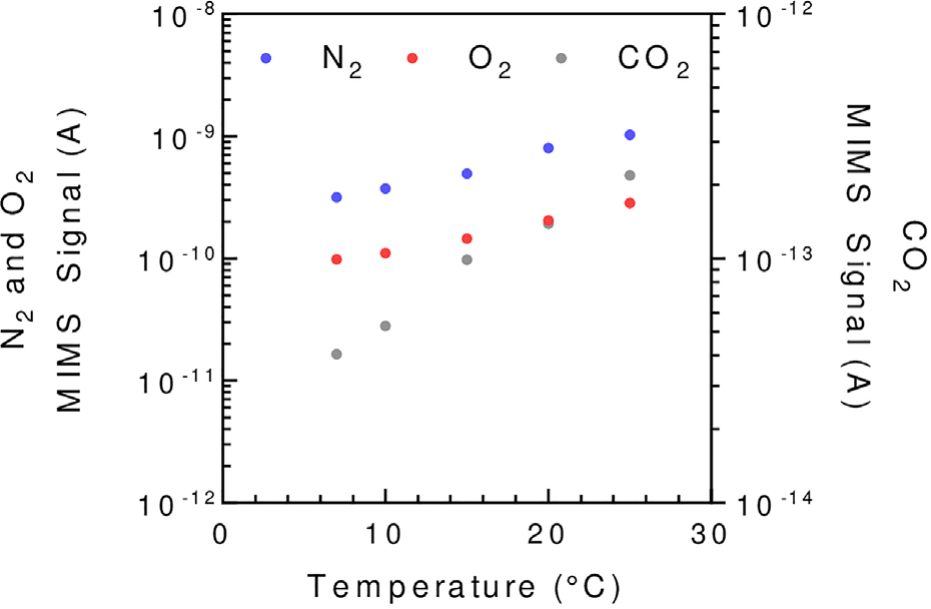
The influence of temperature on the MIMS signal strength illustrated for N_2_, O_2_, and CO_2_. Reaction vessel is filled with *C. reinhardtii* minimal growing medium buffered at pH = 7.2 using 3-(N-morpholino)propane sulfonic acid (MOPS, 20 mM final concentration) and is flushed with air until reaching steady state. A PTFE membrane (13 µm) is used. To avoid solubility issues, the signal shown is normalized by the Bunsen coefficient (see **Methods 1**). The mass spectrometer signal is given in amperes (A).

The membrane is gas permeable and serves as a physical barrier between the liquid phase and the ion source of the mass spectrometer connected by a vacuum line. In the setup described in **Figure 1** the membrane is held at the bottom of the reaction vessel supported by a stainless-steel grid. Note that different membrane inlet setups like microprobes can be used (Kotiaho and Lauritsen, 2002; Konermann et al., 2008). The gas leak through the membrane occurs through a three-step process called permeation: adsorption at the membrane surface, diffusion through the membrane material, and desorption on the other side of the membrane. In this process, diffusion is the limiting factor (Konermann et al., 2008) and is a temperature-dependent process. Thus, membrane permeability is highly influenced by temperature (**Figure 2**), and the reaction vessel must be precisely thermo-regulated (**Figure 1**). The gas leak is crucial since it defines the sensitivity of the method, especially it should remain small compared to the measured reaction rates to limit noise. Gas leakage depends on the membrane properties and is linearly related to the gas concentration inside the vessel (**Methods S1**). Various porous plastic films can be used as a membrane, although polytetrafluoroethylene (PTFE, also known as Teflon) and silicon are the most commonly used (Beckmann et al., 2009; Shevela et al., 2018). The permeability to gases depends on the nature of the membrane. Silicon membranes are more permeable to gases than PTFE membranes (Lloyd et al., 1983) (PTFE membranes can be found on www.hansatech-instruments.com/ or www.ysi.com). The gas leak depends on both membrane surface and thickness, which must be chosen depending on the required sensitivity and on the duration of the experiments to be carried out. The ion source is overly sensitive to water that interferes with ionization and therefore quantification. In this respect, a PTFE membrane has the advantage over silicone of being seven times less permeable to water relatively to N_2_ (Jensen and Cox, 1988), resulting in less noise. A magnetic stirrer is used to maintain algae in suspension and homogenize the gas content of the liquid medium (Lundsgaard et al., 1978). In a case where the stirring occurs on the membrane, the stirring speed must be adjusted to ensure a proper homogenization without compromising membrane integrity. Likewise, a smooth PTFE coated stirring bar can be used to achieve optimal stirring while limiting membrane wear.

### The Vacuum Line and the Cold Trap

The vacuum line connecting the space below the membrane to the ion source of the mass spectrometer is, together with the membrane, a key element to consider when optimizing the response time of the experimental setup. Because of high vacuum (10^−5^–10^−8^ mbar) in the tubing, gases do not flow like a fluid but perform a random walk inside the tube towards the ion source of the mass spectrometer where gases are ionized. During their random walk, gases can adsorb and desorb at the surface of the tubing, thus slowing down the gas flow. The tubing length and cross-section should be optimized to increase the time response without compromising output signal of the mass spectrometer. Limiting length of the tubing decreases the response time of the system both because of limited inner tubing volume and decreased surface adsorption effects of the tubing. On the other hand, while small tubing cross-section favors the time response by limiting the tubing volume, this effect is counter-balanced by an increased probability of molecule absorption on the tubing surface, a too small cross-section decreasing the signal/noise ratio of the setup. We found an inner diameter of ¼ inch to be a good compromise for the type of experiments shown in this paper although smaller tubing down to 116 inch can be suitably used (Konermann et al., 2008).

**Figure 3:**
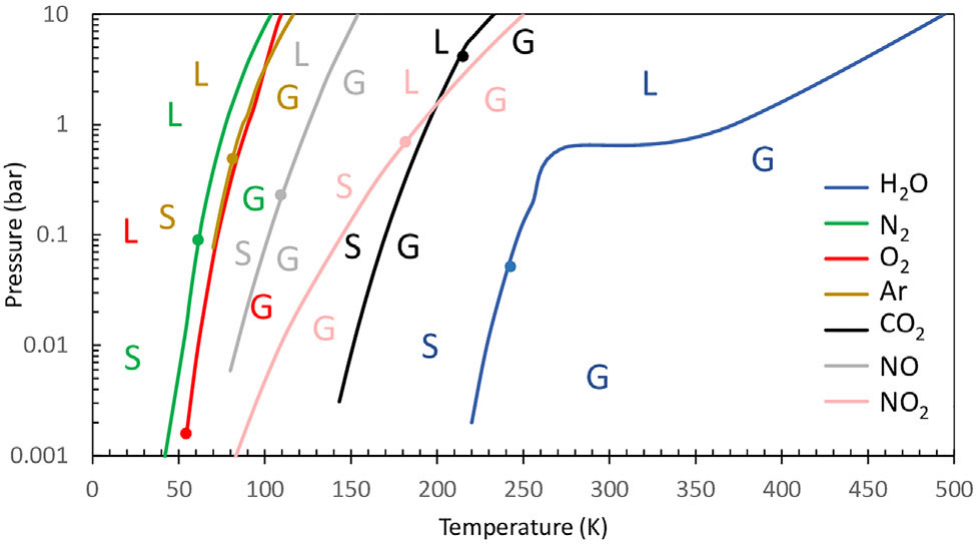
Phase diagram of some gas components of the Earth’s atmosphere. Shown are graphical views of the vapor–liquid and vapor–solid border H_2_O, N_2_, O_2_, Ar, CO_2_, N_2_O, and NO. Dots represent triple points. Letters are placed on the side of the phase diagram, representing gas phase (G), solid phase (S), and liquid phase (L). Shown are data from the gas encyclopedia, Air Liquide, 1976 more data can be found at https://encyclopedia.airliquide.com/.

To limit water vapor entering the mass spectrometer ion source, the vacuum line tubing passes through a cold trap where the water vapor is condensed. Since the cold trap can also condense other gases, the choice of the trap temperature depends on the gas species to be analyzed. For example, cooling the vacuum line with liquid nitrogen (77 K) allows efficiently trapping water but also CO_2_ (**Figure 3**). The condensation points of other main gases [N_2_, O_2_, and argon (Ar)] are much lower than that of water and are usually hard to selectively trap (**Figure 3**). A temperature of 200 K allows selective trapping of water and can be obtained using a mix of ethanol and dry ice in the absence of a cooling unit (Bailleul et al., 2017). The cold trap should be situated as close as possible to the vacuum pump of the mass spectrometer in order to keep its internal pressure as low as possible and limit unintended trapping. For further information on optimization of the membrane inlet system, readers are referred to (Konermann et al., 2008; Beckmann et al., 2009; Shevela et al., 2018).

### Mass Spectrometer

Mass spectrometry is a technique that allows separation and detection of charged molecules in a gas phase depending on their mass over charge (m/z) ratio. During ionization, most reactions produce a single positively charged ion. But most often, the molecule is broken down by ionization, and seldom, double ionization can occur; these effects give rise to two or more fragments. The fragmentation pattern is a signature of the molecule and depends on the ionization method used. In the case of small molecules like gases, the main ion (molecular ion) results from the loss of an electron, other fragments representing a minor part in the fragmentation pattern (**Table 1**). The mass spectrometer, by measuring the signal intensity of detected ions for m/z of interest, enables the determination of gas amounts (**Methods S1**). Any mass spectrometer can be used in a MIMS setup with various ion sources (*e.g.* pulsed ionization, glow discharged ionization) and analyzers (*e.g.* Time Of Flight, Ion Trap) (Johnson et al., 2000). However, magnetic sector or quadrupole mass spectrometers are the most commonly used for gas analysis in a MIMS setup, although quadrupole has the advantage of portability and low price (Ferrón et al., 2016; Bailleul et al., 2017; Chatton et al., 2017).

**Table 1:** Typical mass fragmentation pattern of various gases.

	*H_2_*	*D_2_*	*H_2_O*	*N_2_*	*NO*	*O_2_*	*Ar*	*CO_2_*	*N_2_O*
*Nominal m/z*	2	4	18	28	30	32	40	44	44
*1*	2								
*2*	100	1.59							
*4*		100							
*12*								8.71	
*14*				13.79	7.51				12.91
*15*					2.4				0.1
*16*			0.9		1.5	21.8		9.61	5
*17*			21.22						
*18*			100						
*19*			0.5						
*20*							14.62		
*22*								1.9	
*28*				100				9.81	10.81
*29*				0.74				0.1	0.1
*30*					100				31.1
*31*					0.4				
*32*					0.2	100			
*36*							0.3		
*38*							0.05		
*40*							100		
*44*								100	100
*45*								1.2	0.07
*46*								0.4	

For each gas, shown is the relative amount of m/z in percentage of the maximal signal. Data are extracted from https://webbook.nist.gov/chemistry/. Fragmentations patterns are here given for natural isotopic abundance and therefore include isotopes-specific signal (e.g. m/z = 45 for CO_2_ reflects natural abundance of ^13^CO_2_). Note that the exact fragmentation patterns depend on each ion source and the ionization energy used and must be determined for each instrument.

### Data Management

Because the mass spectrometer is consuming gases, measured variations of gas concentrations must be corrected from the mass spectrometer consumption to determine actual gas exchange rates between the biological sample and the extracellular medium (**Methods S2**). Although this effect has been well described (Berlier et al., 1985; Kotiaho and Lauritsen, 2002; Konermann et al., 2008) its correction has been overlooked in the recent literature. After calculating gas concentrations inside the reaction vessel, gas exchange rates are calculated by correcting from the mass spectrometer consumption. Fluctuations of physical properties of the setup can result in variations of the flux of gas to the mass spectrometer and be an important source of noise. An additional normalization of gas exchange rates to a non-reactive gas (like N_2_ or Ar) can advantageously correct such signal shifts or noise. These artifacts are therefore highly limited although biggest ones remain (**Figure S2**). Note that prior to any experiment, one must ensure the absence of gas leakage in the system (tubing, connections, inlets), which can be done by flushing helium outside the setup and following the m/z = 4 with the mass spectrometer. Details of the calculations have been described in **Methods S1** and **S2**, and we have developed easy-to-run software that allows real time calculation and visualization of MIMS data (Downloadable at: https://github.com/francoisBurlacot/MIMS_Analysis).

## MIMS Usage in Algal Research

**Figure 4:**
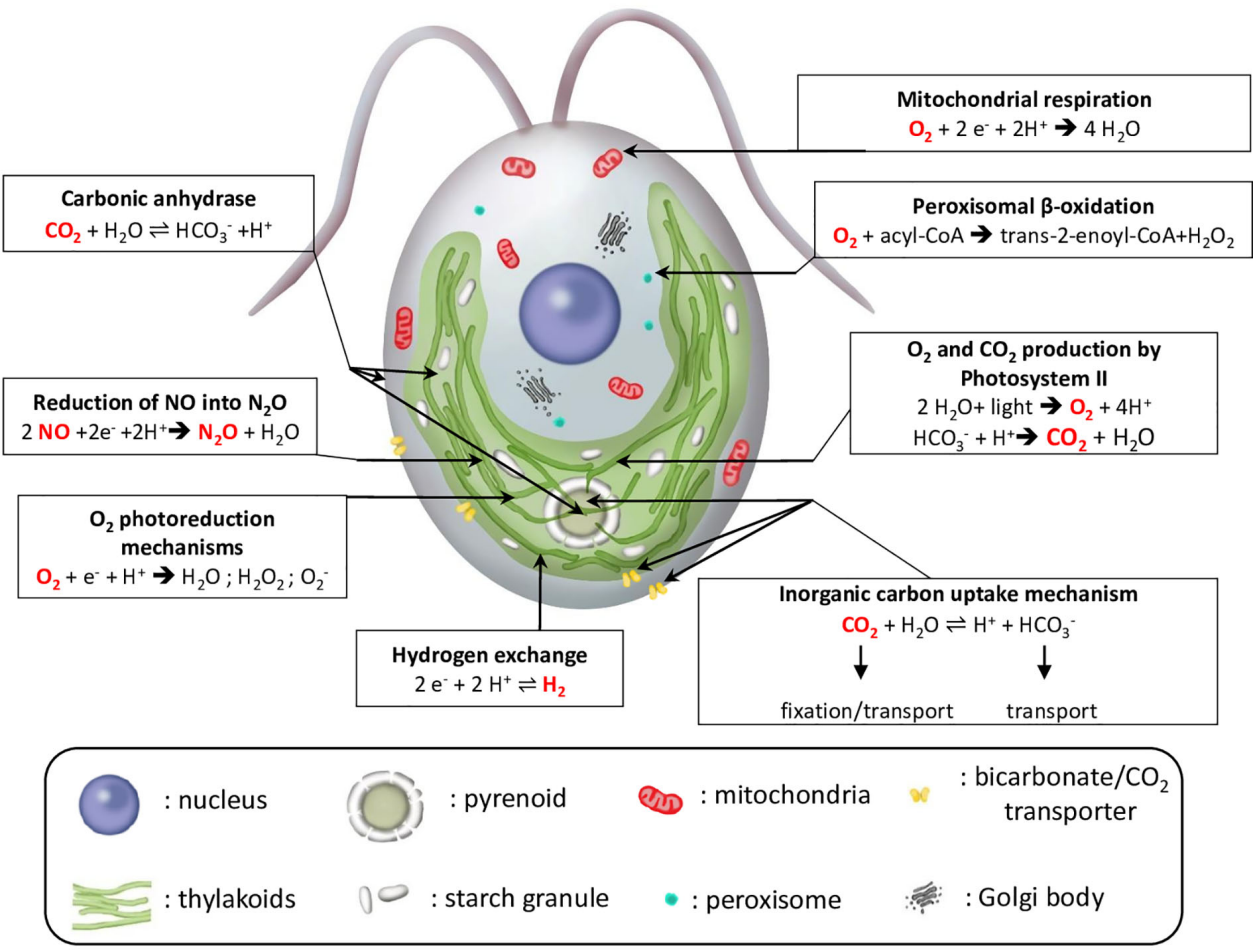
Schematic view of major reactions involving gas exchange in microalgae illustrated for *C. reinhardtii*. Arrows indicate the subcellular localization of the different reactions. For each reaction, gas species that can be measured by MIMS are in red. ADP, adenosine diphosphate; acyl-CoA, acyl-Coenzyme A; e^−^, reducing equivalent; trans-2-enoyl-CoA, trans-2,3-dehydroacyl-Coenzyme A.

MIMS has been initially developed and is still widely used to measure oxygen exchange during photosynthesis. Its usage has been extended to the study of other cellular mechanisms, such as hydrogen production, carbon concentrating mechanisms, and more recently, nitric oxide (NO) photoreduction into N_2_O. We hereby provide some examples of applications of the MIMS in the field of algal biology to measure gas exchanges in the model species *Chlamydomonas reinhardtii* (**Figure 4**).

### Assessment of Photosynthetic Oxygen Exchange

**Figure 5:**
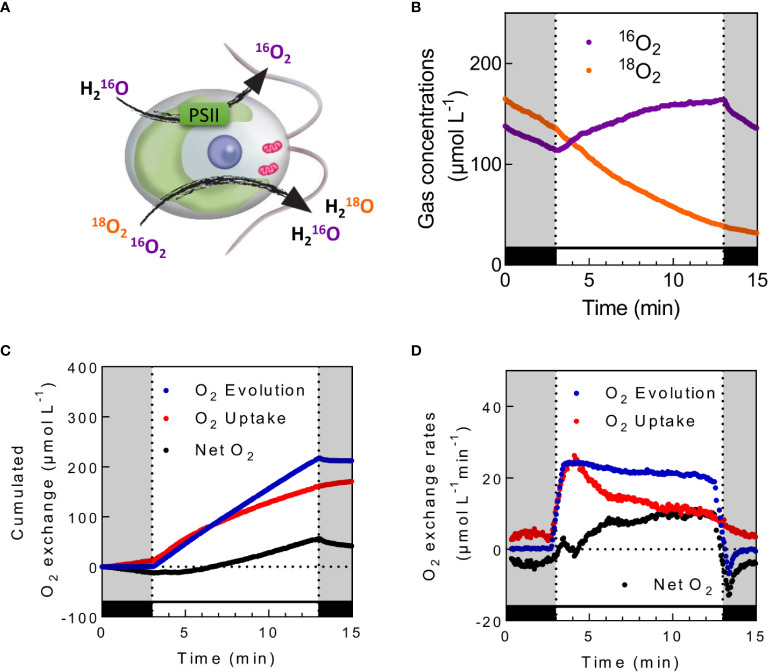
*In vivo* measurements of photosynthetic O_2_ exchange in the presence of ^18^O-labeled O_2_. **(A)**. Schematic view of oxygen exchange illustrated in *C. reinhardtii*. While photosystem II (PSII) produces unlabeled O_2_ from the photolysis of H_2_O, oxygen uptake mechanisms consume both ^18^O-labeled and unlabeled O_2_ **(B)** ^16^O_2_ and ^18^O_2_ concentrations measured in *C. reinhardtii* cells during dark–light transients. **(C, D)**. Calculated cumulated O_2_ exchanges **(C)** and the corresponding O_2_ exchange rates **(D)** for the same experiment. Cells were grown photoautotrophically in air, centrifuged and resuspended in fresh medium at a concentration of 20 µg Chl ml^−1^. Upon addition of 5mM HCO_3_−, ^18^O_2_ was injected inside the cell suspension, and the reaction vessel was closed. After 5 min of dark adaptation, green light was turned on (500 µmol photon m^−2^ s^−1^) for 10 min. Levels of ^16^O_2_ and ^18^O_2_ were recorded at respective m/z = 32 and 36. O_2_ Uptake (red), O_2_ Evolution (blue), and Net O_2_ production (black) were calculated as described; cumulated gas exchange were calculated by directly integrating obtained exchange rates. To limit noise on the exchange rates graphic, data shown in **(D)** are integrated with a sliding average of 30 s wide.

By using ^18^O-enriched O_2_ in illuminated microalgal suspension, Hoch and Kok (Hoch and Kok, 1963) could show that O_2_ can be both produced and consumed during photosynthesis. While O_2_ is produced by PSII from water splitting, O_2_ is simultaneously consumed by different cellular processes (**Figures 4** and **5A**). Practically, the use of highly enriched O_2_ (usually around 99% ^18^O) allows neglecting ^18^O^16^O species, thus measurements of ^16^O^16^O (m/z = 32) and ^18^O^18^O (m/z = 36) are used to determine rates of gross O_2_ evolution (*O_2_ Evolution*) and O_2_ uptake (*O_2_ Uptake*), net O_2_ production rate (*Net O_2_*) being the end result of *O_2_ Evolution* and *Uptake*. Considering that water splitting only produces ^16^O_2_ (the natural abundance of ^16^O being 99.8%, O_2_ is produced from H_2_O at 99.6% as ^16^O_2_) and neglecting isotopic discrimination between ^18^O_2_ and ^16^O_2_ by uptake mechanisms, the following equations modified from (Radmer and Kok, 1976; Peltier and Thibault, 1985b) can be used:

(1) O2 Uptake=vO218×(1+CO218(t)CO218(t))

(2) O2 Evolution=vO216−vO218×CO216(t)CO218(t)

(3) $$Net\;O_{2}=O_{2}\;Evolution+O_{2}\;Uptake$$

where vO218 and vO216 are the gas exchange rates of ^18^O_2_ and ^16^O_2_ respectively; CO218(t) and CO216(t) represent the gas concentrations of ^18^O_2_ and ^16^O_2_ respectively (see **Methods S1** and **S2**). Typical patterns of ^18^O_2_ and ^16^O_2_ concentration, gross O_2_ evolution, O_2_ uptake, and O_2_ net production rates as well as cumulated O_2_ exchanges measured in *C. reinhardtii* cells during a dark to light transient are shown in **Figures 5B–D**.

Measuring O_2_ exchange by MIMS in microalgae and cyanobacteria allowed dissecting molecular players involved in the O_2_ uptake process, initially by using various inhibitors or characterizing the dependency on O_2_ or CO_2_ concentrations (Badger et al., 2000). (Radmer and Kok, 1976) early proposed that a highly efficient O_2_ photoreduction process was present in cyanobacteria and microalgae. The persistence of mitochondrial respiration in the light was shown to contribute (Peltier and Thibault, 1985b), and the existence of a CO_2_-dependent O_2_ uptake component was evidenced (Badger, 1985; Sültemeyer et al., 1987). With the development of genetic approaches, the nature and contribution of the different players were better characterized. For example, the contribution of mitochondrial respiration to O_2_ uptake is enhanced in the absence of the proton gradient regulation like 1 (PGRL1)-mediated cyclic electron flow (CEF), thus showing the functional complementarity between these pathways in the production of intra-cellular ATP (Dang et al., 2014). Redox communication between chloroplast and peroxisome (Kong et al., 2018) or with mitochondria in diatoms (Bailleul et al., 2015) was also evidenced from O_2_ exchange measurements. The involvement of Flavodiiron proteins (Flvs) in light-dependent O_2_ uptake was further established in cyanobacteria (Helman et al., 2003; Allahverdiyeva et al., 2013), and more recently in microalgae (Chaux et al., 2017). MIMS is nowadays widely used to understand the fate of the photosynthetic electron flow in various environmental conditions and mutant of cyanobacteria (Ermakova et al., 2016; Boatman et al., 2018; Luimstra et al., 2019), microalgae (Fisher and Halsey, 2016), or coral reef symbiosis (Einbinder et al., 2016).

If laboratory studies performed on model species allowed recognizing O_2_ photoreduction as a major alternative photosynthetic electron sink crucial for cell acclimation to various environmental conditions (Curien et al., 2016), the relative contribution of different O_2_ uptake mechanisms in natural environments remains largely unexplored (Bailleul et al., 2017). Despite its performance in measuring gross photosynthesis, MIMS has long remained a cumbersome technique, not suitable for field experiments. On the other hand, chlorophyll fluorescence measurements using pulse-amplitude modulated (PAM) fluorimeter have been widely used for estimating PSII yield in natural environments such as in the ocean (Falkowski and Kolber, 1995). Chlorophyll fluorescence however, faces some limitations when used to determine gross O_2_ production since the estimation of electron transport rates requires the measurement of cell absorbance (Genty et al., 1989; Godaux et al., 2015), which is difficult to realize in outdoor conditions. Recently, the miniaturization of mass spectrometers allowed *in situ* measurements of O_2_ exchange in phytoplankton from the north Pacific (Ferrón et al., 2016) or in planktonic blooms from the north Atlantic (Bailleul et al., 2017), starting thus a new era for expanding research obtained in the laboratory on model species to species in their natural environments.

Indeed, fluorescence measurements have often been used together with MIMS CO_2_ and O_2_ exchange measurements to study the link between CO_2_ fixation and non-photochemical quenching of chlorophyll fluorescence (Sültemeyer et al., 1989; Fratamico et al., 2016; Ware et al., 2020). Furthermore, in conditions where the use of ^18^O_2_ is not possible (*e.g.* anaerobiosis), coupling PSII quantum yield with gas exchanges has recently allowed determining gross O_2_ production and inferring the existence of an O_2_ uptake process (Burlacot et al., 2018).

### Hydrogen Production and Hydrogenase Activity Measurements

In microalgae and cyanobacteria, hydrogenases (H_2_ases) catalyze the reversible formation of hydrogen (H_2_) by direct reduction of protons (H^+^). In microalgae, the electron donor to the [Fe–Fe] H_2_ase is ferredoxin (Florin et al., 2001), which can be reduced by the photosynthetic electron transport chain or by fermentative pathways (Catalanotti et al., 2013). Experimentally, H_2_ production can be measured by different techniques, including modified O_2_ electrodes (Wang, 1980; Godaux et al., 2015), gas chromatography (Hunt and Smith, 1961; Nagy et al., 2018), nuclear magnetic resonance spectroscopy (NMR) (Xu et al., 2016; Manz et al., 2017), or MIMS. Among these techniques, MIMS and modified O_2_ electrodes allow *in vivo* quantitative measurement of hydrogen in a time-resolved manner. Under natural conditions, hydrogen photoproduction by microalgae is a transient phenomenon, generally considered as a safety valve avoiding over-reduction of PSI electron acceptors under anaerobiosis (Ghysels et al., 2013). Hydrogen photoproduction is limited by the O_2_ sensitivity of H_2_ase, O_2_ being produced by PSII during illumination (Erbes et al., 1979). When using experimental conditions maintaining anaerobic conditions (thus limiting the H_2_ase inhibition) like sulfur deprivation (Melis et al., 2000), low illumination (Liran et al., 2016) or O_2_ quenchers like glucose and glucose oxidase/catalase (Godaux et al., 2015), a limitation of the supply of electrons to the H_2_ase can be evidenced. The use of MIMS and of various *C. reinhardtii* mutants allowed identifying biological bottlenecks limiting the supply of electrons to the H_2_ase (Tóth and Yacoby, 2019; Burlacot et al., 2020a). Lately, MIMS was used in the development of a very promising H_2_ photoproducing protocol using flashing light as the light source in photoautotrophic self-anaerobic conditions (Kosourov et al., 2018).

MIMS has early been used for *in vitro* and *in vivo* measurements of H_2_ase activity (Jouanneau et al., 1980; Vignais et al., 1982; Berlier et al., 1985). In the presence of H_2_, H_2_ase spontaneously splits H_2_ (Hoberman and Rittenberg, 1943; Rittenberg and Krasna, 1955), forming one proton with the reversible reaction:

(4) $$Hyd+H_{2}⇌Hyd:H^{-}+H^{+}$$

where Hyd is the binding site of H_2_ase. When supplying deuterium (D_2_), HD is formed during the back reaction (6) in the presence of protons in the reaction mixture:

(5) $$Hyd:D^{-}+H^{+}⇌Hyd+HD$$

**Figure 6:**
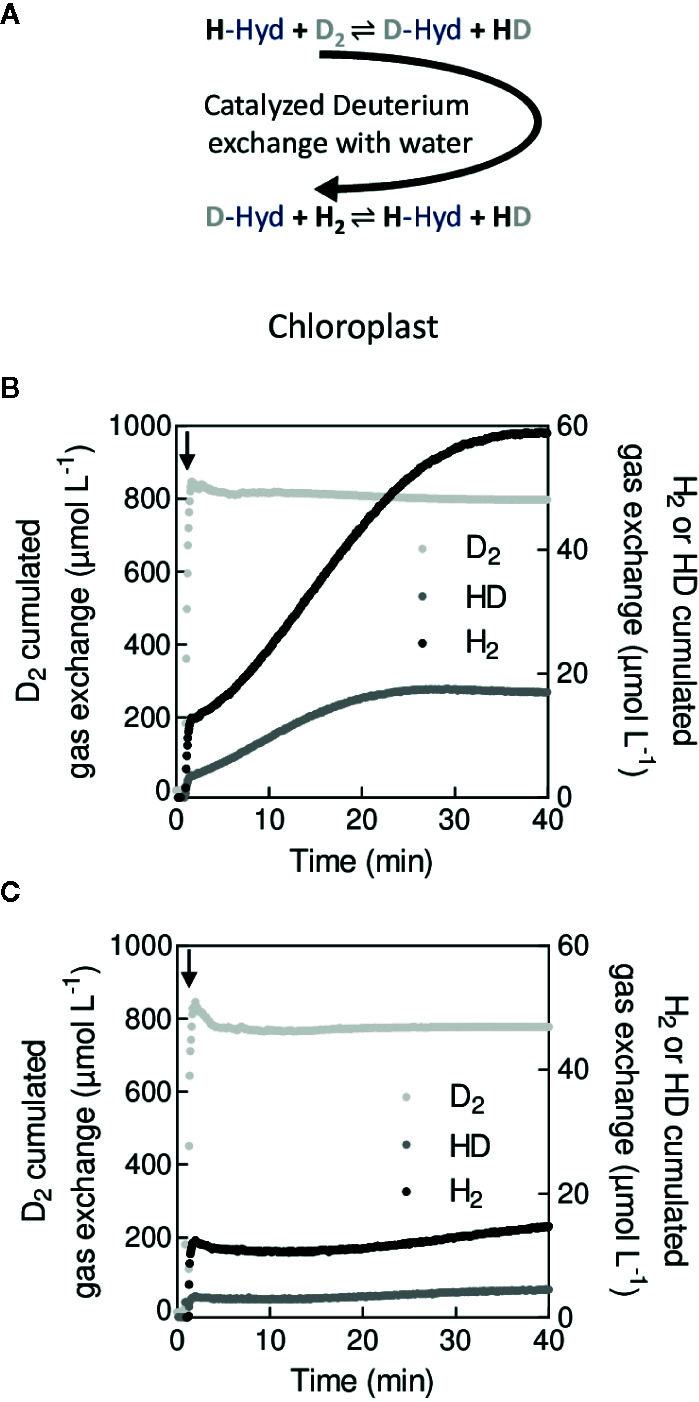
*In vivo* measurement of the hydrogenase activity by H/D exchange **(A)**. Principle of H/D exchange. In the presence of labeled hydrogen (D_2_), the labeled deuterium (D^+^) is exchanged with protons (H^+^) at the catalytic site of H_2_ase (-Hyd). **(B)**. Cumulated gas exchange of D_2_, HD, and H_2_ in wild type cells after 1 h anaerobiosis with induced hydrogenase. **(C)**. Cumulated gas exchange of D_2_, HD, and H_2_ in wild type cells after 1 min of anaerobiosis without induction of hydrogenase. For **(B, C)**, cell suspension of *C. reinhardtii* was maintained in anaerobiosis for 1 min **(B)** or 1 h **(C)** before t = 0. At t = 1 min (black arrow), D_2_ was bubbled for a few seconds before the reaction vessel was closed and H/D exchange recorded.

This reaction directly depends on the turnover rate of H_2_ases (*i.e.* H_2_ase activity) (Vignais, 2005). In the absence of H_2_ production or uptake, following kinetics of D_2_, HD and H_2_ with MIMS allows measuring the H_2_ase activity (H_2_ase activity) by the H/D exchange rate (V_exch_):

(6) $$H_{2}ase\;\mathrm{activity}=V_{exch}(t)=\frac{1}{\tau }(2v_{H_{2}}(t)+v_{HD}(t))$$

with

(7) $$\tau =\frac{C_{D_{2}}(t)+\frac{C_{HD}(t)}{2}}{C_{D_{2}}(t)+C_{H_{2}}(t)+C_{HD}(t)}$$

where $C_{D_{2}}(t)$,$C_{H_{2}}(t)$ and $C_{HD}(t)$ are the concentrations of D_2_, H_2_ and HD respectively and $v_{H_{2}}(t)$,$v_{HD}(t)$ being the gas exchange rates of H_2_ and HD, respectively (see **Methods S1** and **S2**) (Cournac et al., 2004). A few microalgal species such as *C. reinhardtii* harbor H_2_ases (Burlacot and Peltier, 2018) under anaerobic conditions. Measuring the H/D exchange allowed monitoring of H_2_ase induction or inhibition *in vivo* (Vignais et al., 2002; Tolleter et al., 2011; Burlacot et al., 2018). Typical patterns of D_2_, H_2_ and HD exchange measured upon injection of D_2_ before and after induction of H_2_ase in *C. reinhardtii* are shown on **Figure 6**. Note that in conditions where H_2_ase produces H_2_ during illumination the H_2_ase activity, needs to be corrected from the increase in total hydrogen species (H_2_, HD, D_2_) (Cournac et al., 2004). Although gas chromatography or NMR has also the potential to differentiate D_2_, HD and H_2_ (Hunt and Smith, 1961; Xu et al., 2016), MIMS allows performing such measurements *in vitro* and *in vivo* in a time resolved manner. If H/D exchange measurements using a MIMS allow determining the catalytic constant of H_2_ase, it can also be used to determine the resistance of gas diffusion between the active H_2_ase site and the reaction medium *in vitro* (Leroux et al., 2008). *In vitro* H/D exchange measurements have been used to study enzymatic properties of native H_2_ase (Abou Hamdan et al., 2012; Gauquelin et al., 2018), including O_2_-tolerant H_2_ases (Liebgott et al., 2011), as well as H_2_ases modified by site-directed mutagenesis in order to limit O_2_ diffusion to the active site (Cano et al., 2014). H_2_ photoproduction by microorganisms has recently regained huge interest for biofuel production due to recent improvements in strains and experimental protocols (Tóth and Yacoby, 2019). The use of MIMS should help in evaluating the upcoming combination of newly developed H_2_ production protocols (Kosourov et al., 2018; Nagy et al., 2018) and previously characterized mutants photo-producing more H_2_ (Tolleter et al., 2011; Eilenberg et al., 2016; Burlacot et al., 2018; Ben-Zvi et al., 2019). This has recently started using *flv* mutants (Jokel et al., 2019) and is promising for future bio H_2_ production developments.

### Assessing NO and N_2_O Gas Exchange

**Figure 7:**
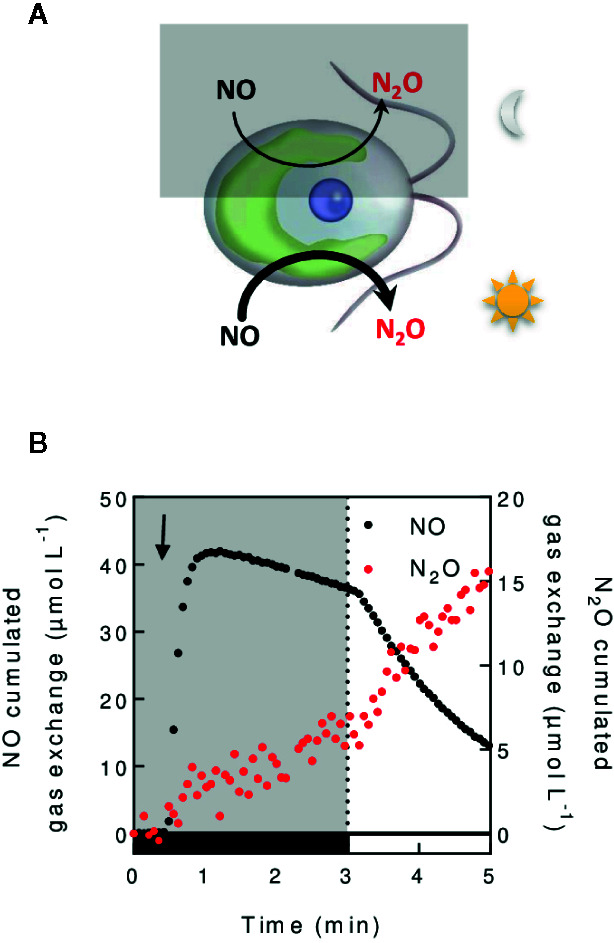
In vivo measurement of NO reduction into N_2_O. **(A)**. Schematic N_2_O production mechanisms illustrated in *C. reinhardtii*. Nitric oxide (NO) is reduced in the chloroplast both in a light dependent and independent manner (Burlacot et al., 2020b). **(B)**. Cumulated gas exchange of NO and N_2_O in *C. reinhardtii* cells grown autotrophically (100 µg chlorophyll. ml^−1^) during a dark to light transition. Glucose oxidase/catalase and glucose are added to the algal suspension to reach anaerobiosis. After injection of a NO-saturated water solution in the cell suspension (black arrow), NO and N_2_O exchange is measured as described in (Burlacot et al., 2020b).

On Earth, 6% of the radiative forcing is due to N_2_O (IPCC, 2013), whose greenhouse effect is 300 times that of CO_2_. N_2_O is produced from the reduction of nitric oxide (NO) by bacteria (Goreau et al., 1980), fungi (Maeda et al., 2015), and microalgae (Guieysse et al., 2013). The measurement of N_2_O in a time resolved manner can help understand the dynamics of N_2_O formation in the environment or in isolated organisms. In green microalgae, MIMS has recently been used to dissect the molecular mechanisms involved in the conversion of NO into N_2_O (**Figure 7**) (Burlacot et al., 2020b). In practical terms, the detection of N_2_O requires specific calculations because its mass spectrum overlaps that of CO_2_ (see **Methods S1**). However, due to a relatively high detection limit for N_2_O (around 18 µM) (Chatton et al., 2017), MIMS is not suitable for detecting low N_2_O amounts, such as in water in equilibrium with ambient air (4.3 µM) (IPCC, 2013).

NO is an important intracellular signaling molecule in algae like in most living organisms. While NO is crucial for growth of *C. reinhardtii* under anaerobiosis and for its acclimation to nitrogen, sulfur or phosphate deprivation (Hemschemeier et al., 2013; Wang and Spalding, 2014; De Mia et al., 2019; Filina et al., 2019), mechanisms of NO production remain blurry. The use of MIMS in algae fed with nitrates or nitrites, allowed to evidence the role of NO reduction mechanisms in the regulation of NO homeostasis (Burlacot et al., 2020b). Following NO production can also be based on imaging fluorescent chemical probes specifically reacting with NO (Li and Wan, 2015) or on the use of specific electrodes (Csonka et al., 2015). However, MIMS by supplying quantitative, time resolved, and stable measurements of NO (Bethke et al., 2004; Conrath et al., 2004), with further simultaneous measurement of NO reduction products such as N_2_O, should help deciphering mechanisms participating in NO homeostasis.

### Inorganic Carbon Affinity in Microalgae and Carbonic Anhydrase Activity

**Figure 8:**
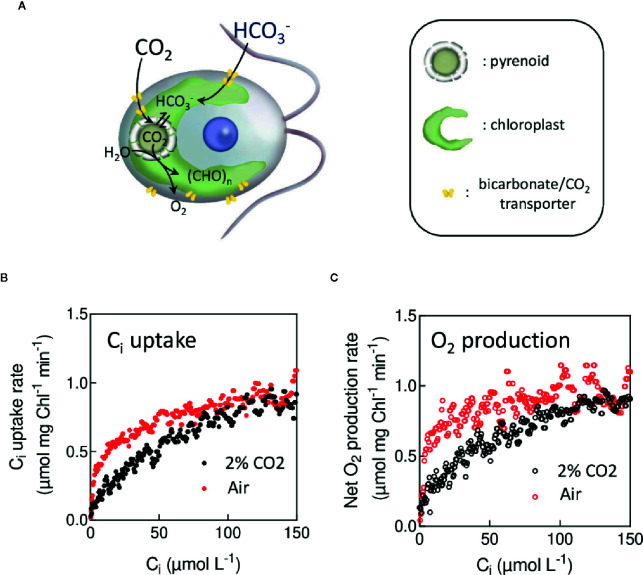
*In vivo* measurement of the apparent affinity of photosynthesis for CO_2_ during the induction of the Carbon Concentrating Mechanisms. **(A)**. Schematic view of the carbon transport mechanism in *C. reinhardtii*. In the presence of a CCM, inorganic carbon (C_i_) is actively transported, thus increasing the CO_2_ concentration at the CO_2_ fixation site. **(B)**. C_i_ uptake rates measured at different C_i_ concentrations during its depletion in *C. reinhardtii* cells grown in 2% CO_2_ in air (in red) or in air levels of CO_2_ (in black). **(C)**. Net O_2_ production rates depending on C_i_ during the same experiments as in **(B)**. *C. reinhardtii* cells were grown either at air level of CO_2_ or 2% CO_2_, growing medium; cell sampling was the same as described in **Figure 2**. After sampling and resuspention in fresh medium, green saturating light (3,000 µmol photon m^−2^ s^−1^) was turned on, and O_2_ production and C_i_ uptake were recorded during the depletion of C_i_. With this technique, the apparent affinity for C_i_ of photosynthesis measured by net O_2_ production in cells with an active CCM (K_1/2_ = 5 µM) is 10 times higher than the one measured when the CCM is not induced (K_1/2_ = 50 µM).

When grown under low CO_2_ concentration, many microalgae or cyanobacteria induce an active import of inorganic carbon (C_i_ = CO_2_ and HCO_3_^−^) (**Figure 8A**) from the extracellular medium to the active site of CO_2_ fixation (Badger et al., 1980; Badger and Andrews, 1982; Sültemeyer et al, 1991; Giordano et al., 2005; Reinfelder, 2011). This mechanism, called Carbon Concentrating Mechanism (CCM), principally operates by concentrating HCO_3_^−^ inside cells (Price et al., 2007). In microalgae, HCO_3_^−^ is converted into CO_2_ inside the pyrenoid, at the vicinity of the carbon-fixing enzyme (RuBisCO) thus increasing the local CO_2_ concentration (**Figure 8A**) (Mackinder, 2018). The CCM ensures high C_i_ fixation rates by photosynthesis under low C_i_ concentration (Badger et al., 1980). When active, the CCM results in an increased apparent affinity of photosynthesis for C_i_, which can be assessed by measuring O_2_ production rates at various C_i_ concentrations, either using an O_2_ electrode (Badger et al., 1980) or a MIMS (Sültemeyer et al., 1993). MIMS has the advantage of simultaneously measuring O_2_ and CO_2_. Since the CO_2_ decreases during the time course of the experiment due to the activity of photosynthesis, it is possible to determine in one single experiment net O_2_ or CO_2_ exchange rates at different CO_2_ concentrations ($v_{O_{2}}(C_{CO_{2}}(t))$ or $v_{O_{2}}(C_{CO_{2}}(t))$) during CO_2_ fixation by photosynthesis (Douchi et al., 2019). **Figures 8B, C** show typical experiments in which CO_2_ uptake and O_2_ production rates have been determined as a function of the CO_2_ concentration in air-grown and CO_2_-grown cells of *C. reinhardtii*. Note that in such experiments the cell concentration must be kept low enough to ensure that photosynthetic gas exchange kinetics are sufficiently slow as compared to the response time of the MIMS setup. Using this technique (**Figure 8**), the apparent affinity for C_i_ of photosynthetic O_2_ production of cells with an active CCM is about 10 times higher than in cells with no active CCM as previously reported (Badger et al., 1980; Sültemeyer et al., 1998).

During the induction of CCM, carbonic anhydrases (CAs) are also induced and catalyze the reversible hydration of CO_2_ by water which is summarized in reaction (8):

(8) $$CO_{2}+H_{2}O⇌HCO_{3}^{-}+H^{+}$$

In microalgae, different CA isoforms are present in the different cellular compartments, thus limiting the disequilibrium between transported and consumed C_i_ species (**Figure 4**) (Sültemeyer et al., 1990; Mackinder et al., 2017). Inside the pyrenoid, CA rapidly converts HCO_3_^−^ into CO_2_, the substrate of RuBisCO, thus ensuring a high CO_2_ concentration at the catalytic site of the enzyme (Mackinder, 2018).

When their CCM is active, cyanobacteria and microalgae can take both CO_2_ and HCO_3_^−^ in the medium (Sültemeyer et al., 1991; Badger et al., 1994). A way to assess the net flux of both species is to use the disequilibrium between inorganic species (see equation 8) induced by the preferential uptake of one species. Note that this approach is not possible in the presence of extracellular CA, which has limited its usage in microalgae (Badger et al., 1994; Palmqvist et al., 1994). By comparing net O_2_ production (proportional to the overall net C_i_ uptake) to the net CO_2_ uptake and knowing the uncatalyzed rate of CO_2_ and HCO_3_^−^ interconversion (8), it is possible to calculate the net HCO_3_^−^ uptake (Badger et al., 1994). Using the disequilibrium method with MIMS has shown that HCO_3_^−^ was preferentially taken during CCM in cyanobacteria (Sültemeyer et al., 1998).

**Figure 9:**
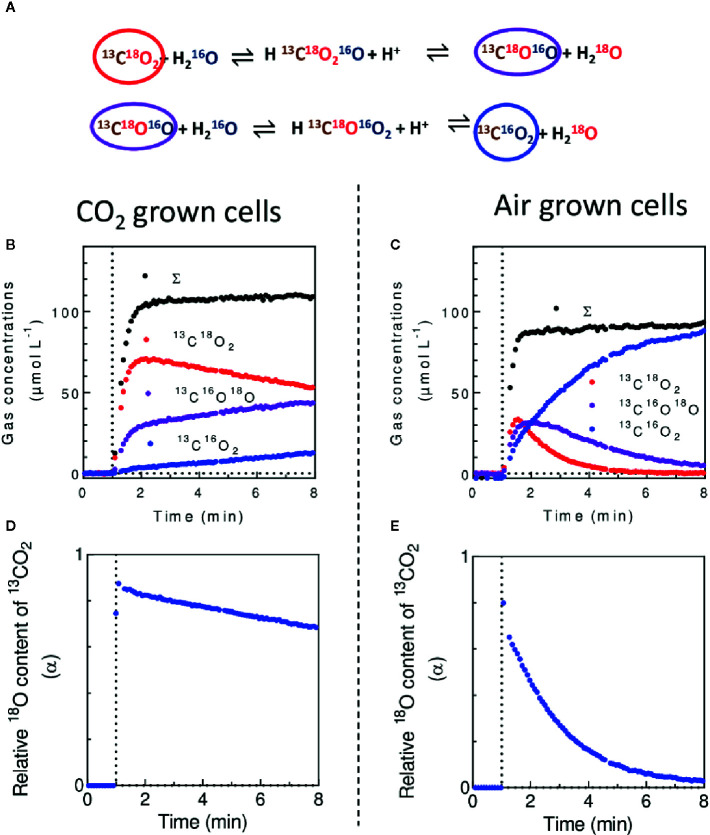
*In vivo* measurement of carbonic anhydrase (CA) activity. **(A)**. Cascade of reactions leading to the unlabeling of ^18^O-enriched CO_2_ in solution in H_2_O. **(B, C)**. Concentrations of ^13^C^16^O_2,_^13^C^18^O^16^O, ^13^C^18^O_2_ and total ^13^C-labeled carbon form (Σ) upon injection of ^13^C^18^O_2_ in a suspension of *C. reinhardtii* cells cultured in 2% CO_2_ **(B)** or at air level of CO_2_ **(C)**. **(D, E)**. Isotopic ^18^O content (*α*) of ^13^CO_2_ during experiments of **(B, C)** respectively. ^13^C and ^18^O-enriched CO_2_ was used to avoid the mass spectrometric background on m/z = 44 due to naturally present CO_2_, therefore enhancing the signal to background ratio (Radmer and Kok, 1976). ^13^C^18^O_2_ was injected after 1 min of darkness at 0.1 mM final concentration (vertical dotted line).

In the presence of doubly ^18^O-labeled CO_2_, the CA activity which catalyzes the exchange of oxygen isotopes between CO_2_ and H_2_O results in a progressive dilution of ^18^O from CO_2_ (Gerster, 1971) (**Figure 9A**). To limit the background level of m/z = 44 (^12^CO_2_) due to naturally present CO_2_ in the algal suspension, the assay can be done using ^18^O-enriched ^13^CO_2_ (Radmer and Kok, 1976), following the m/z = 45 (^13^C^16^O_2_), 47 (^13^C^18^O^16^O) and 49 (^13^C^18^O_2_). After injection of ^13^C^18^O_2_ (supplied as H^13^C^18^O_3_^−^ in a buffered reaction medium) a typical pattern of the progressive unlabeling of ^13^CO_2_ in *C. reinhardtii* cells is shown (**Figures 9B–E**). The isotopic content of ^18^O in ^13^CO_2_ during the progressive unlabeling is given by:

(9) α=2×CC13O218(t)+CC13O18O16(t)CC13O218(t)+CC13O18O16(t)+CC13O216(t)

where CC13O218(t), CC13O18O16(t) and CC13O216(t) are the concentrations of ^13^C^18^O_2_, ^13^C^18^O^16^O and ^13^C^16^O_2_ respectively (**Methods 1**). On purified CA, the isotopic enrichment decays exponentially:

(10) $$\alpha =a_{1}\times e^{-\theta t}$$

where *θ* is the rate constant of the exchange of ^18^O with water (Gerster, 1971; Silverman, 1982). Note that in some cases like a concomitant use of ^18^O-labeled O_2_, the presence of ^18^O and C^16^O_2_ in the ion source of the mass spectrometer can spontaneously generate C^18^O^16^O which needs to be corrected (Cournac et al., 1993). *In vivo*, a CA isotope exchange assay measures the global contribution of all CAs present in the biological sample with an additional effect due to diffusion/transport of C_i_ through the membranes (Sültemeyer and Rinast, 1996; Tolleter et al., 2017). When a periplasmic CA is present, as it is the case in *C. reinhardtii* cells with an active CCM, its activity dominates the exchange kinetics (Sültemeyer and Rinast, 1996). MIMS is so far the most reliable method for CA activity measurements in biological samples and is particularly suitable for *in vivo* measurements on algae (Dang et al., 2014; Benlloch et al., 2015; Tolleter et al., 2017), cyanobacteria (Whitehead et al., 2014), corals (Tansik et al., 2015), and plants (Peltier et al., 1995; Clausen et al., 2005; Burén et al., 2011; Benlloch et al., 2015). *In vitro*, the use MIMS and H^13^CO_3_^−^ has also allowed unraveling the existence of a light-induced CO_2_ production by the PSII (Koroidov et al., 2014; Shevela et al., 2020).

## Future Developments and Perspectives

Although MIMS is more than 50 years old, its usage has only recently become popular (Ketola and Lauritsen, 2016). The recent development in setups and analytical protocols and its popularity helped pushed the limits of our knowledge in the biology of photosynthetic microorganisms at various scales (Burlacot et al., 2020a).

Gas exchange measurements, chlorophyll fluorescence (Maxwell and Johnson, 2000), and electrochromism measurements (Bailleul et al., 2010) are the three main tools available to measure photosynthetic activity in microalgae and cyanobacteria on intact organisms. Simultaneous measurements of gas exchange by MIMS and chlorophyll fluorescence have allowed for correlating energy dissipation processes with CO_2_ and O_2_ photoreduction occurring during photosynthesis (Sültemeyer et al., 1989; Burlacot and Peltier, 2018; Ware et al., 2020). However, our understanding is still limited by the functional redundancy of many mechanisms and their interaction with cryptic mechanisms such as cyclic electron flow, which are not easily experimentally accessible. Further coupling of these methods, allowing for instance parallel measurement of gas exchange by MIMS and cyclic electron flow by electrochromism, together with an increased accessibility to genetic resources, should provide in the future new insights on how the main photosynthetic processes are regulated and interact during acclimation to various environmental situations.

In the perspective of large-scale biofuel production by microalgae or cyanobacteria, recent research has focused on the design and use of photobioreactors coupled to MIMS for the analysis of volatile compounds of interest. These setups have been used to measure real time productions of H_2_ (Tamburic et al., 2011; Zhang et al., 2015) or ethylene (Zavřel et al., 2016) during medium and long-term cultivation of microalgae or cyanobacteria. In the future, MIMS could be used to study and optimize the production in photobioreactors of volatile hydrocarbons by engineered photosynthetic cells.

Further miniaturization and decreasing prices of mass spectrometers should enable an even larger number of laboratories to have access to MIMS in the future, thus accelerating our understanding of how photosynthetic microorganisms impact the atmosphere of our planet (Burlacot et al., 2020a). The use or application of MIMS in the field opens a new era of evaluating the occurrence and ecological relevance of molecular mechanisms in natural environment.

## Data Availability Statement

All datasets presented in this study are included in the article/**Supplementary Material**.

## Author Contributions

AB and GP designed the illustrating experiments. AB performed the experiments. AB and FB designed the software. FB developed the software with supervision from AB. AB, YL-B and GP wrote the manuscript.

## Funding

This work was supported by the French Agence Nationale de la Recherche (ANR) projects OTOLHYD and PHOTOALKANE. AB is a recipient of a CEA international PhD studentship (Irtelis).

## Conflict of Interest

The authors declare that the research was conducted in the absence of any commercial or financial relationships that could be construed as a potential conflict of interest.
