# Corrigendum: Membrane Inlet Mass Spectrometry: A Powerful Tool for Algal Research

**DOI:** 10.3389/fpls.2020.631667

**Published:** 2020-12-22

**Authors:** Adrien Burlacot, François Burlacot, Yonghua Li-Beisson, Gilles Peltier

**Affiliations:** Aix Marseille Univ, Commissariat à l'énergie Atomique et aux énergies Alternatives (CEA), Centre National de la Recherche Scientifique (CNRS), Institut de Biosciences et Biotechnologies d'Aix- Marseille (BIAM), CEA Cadarache, Saint Paul-Lez-Durance, France

**Keywords:** gas exchange, photosynthesis, carbonic anhydrase, CO_2_ concentrating mechanism, O_2_ evolution, H_2_ production, microalgae, cyanobacteria

In the original article, in the paragraph on the “Assessment of Photosynthetic Oxygen Exchange,” we defined the O_2_ uptake rate with a negative value when oxygen is consumed. Although it has been historically the first way to define it (Hoch and Kok, 1963), it makes more sense to use a positive value as adopted later by Radmer and Kok (1976) because a negative uptake would mean the usage of a double negative which implies production.

Therefore, Equations (1), (2), and (3) should be read;

(1) $$\begin{array}{r} O_{2}\;Uptake_{\;}={-v}_{{}_{\;}^{18}O_{2}}\times \left(1+\frac{C_{{}_{\;}^{16}O_{2}}\left(t\right)}{C_{{}_{\;}^{18}O_{2}}\left(t\right)}\right) \end{array}$$

(2) $$\begin{array}{r} {O_{2}\;Evolution}_{\;}=v_{{}_{\;}^{16}O_{2}}-v_{{}_{\;}^{18}O_{2}}\times \frac{C_{{}_{\;}^{16}O_{2}}\left(t\right)}{C_{{}_{\;}^{18}O_{2}}\left(t\right)} \end{array}$$

(3) $$\begin{array}{r} {Net\;O_{2}}_{\;}=O_{2}\;Evolution-O_{2}\;Uptake \end{array}$$

As a consequence, the plots shown on the original Figure 5 have been replaced by the attached Figure 5.

**Figure 5:**
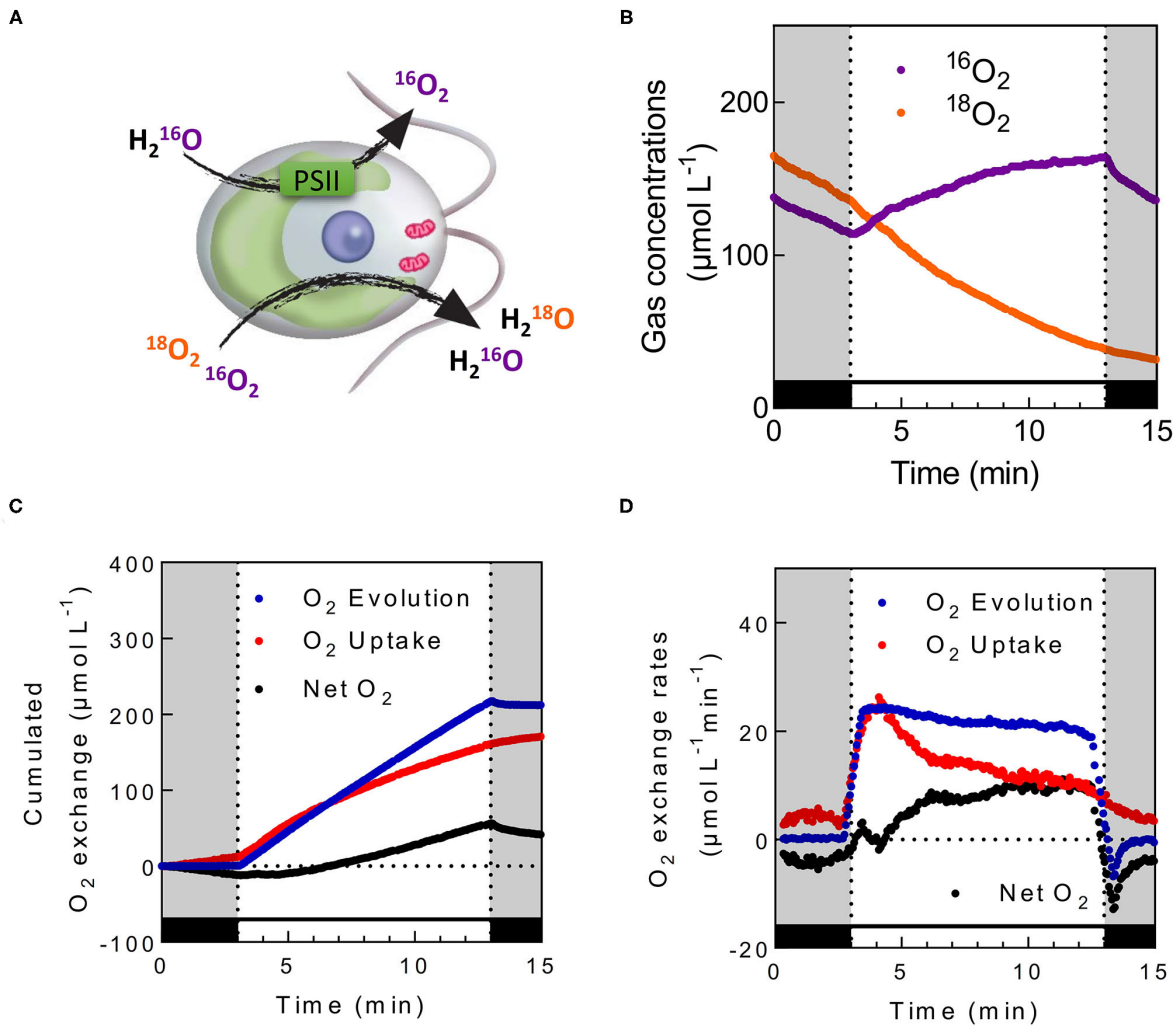
*In vivo* measurements of photosynthetic O_2_ exchange in the presence of ^18^O-labeled O_2_. **(A)** Schematic view of oxygen exchange illustrated in *C. reinhardtii*. While photosystem II (PSII) produces unlabeled O_2_ from the photolysis of H_2_O, oxygen uptake mechanisms consume both ^18^O-labeled and unlabeled O_2_ **(B)** ^16^O_2_ and ^18^O_2_ concentrations measured in *C. reinhardtii* cells during dark–light transients. **(C,D)** Calculated cumulated O_2_ exchanges **(C)** and the corresponding O_2_ exchange rates **(D)** for the same experiment. Cells were grown photoautotrophically in air, centrifuged and resuspended in fresh medium at a concentration of 20 μg Chl ml^−1^. Upon addition of 5 mM HCO${}_{3}^{-}$, ^18^O_2_ was injected inside the cell suspension, and the reaction vessel was closed. After 5 min of dark adaptation, green light was turned on (500 μmol photon m^−2^ s^−1^) for 10 min. Levels of ^16^O_2_ and ^18^O_2_ were recorded at respective m/z = 32 and 36. O_2_ Uptake (red), O_2_ Evolution (blue), and Net O_2_ production (black) were calculated as described; cumulated gas exchange were calculated by directly integrating obtained exchange rates. To limit noise on the exchange rates graphic, data shown in **(D)** are integrated with a sliding average of 30 s wide.

The authors would like to thank Dr. Duncan Fitzpatrick for highlighting this problem and for suggesting changes to increase clarity of the article.

The authors apologize for this error and state that this does not change the scientific conclusions of the article in any way. The original article has been updated.
