# Supplementary material for: Membrane Inlet Mass Spectrometry: A Powerful Tool for Algal Research
Source: Front Plant Sci. 2020 Sep 4;11:1302. doi: 10.3389/fpls.2020.01302 (PMC7500362; doi:10.3389/fpls.2020.01302)
Supplement: Supplementary file 1 [file DataSheet_1.pdf]

Methods S1 Calculating gas concentrations

The amperometric signal generated by the mass spectrometer for each m/z ratio is linearly correlated to the gas concentration inside the reaction vessel. We describe here the general principle in the case of O_2 (m/z=32), which also applies to other gases of interest. The m/z=32 amperometric signal for a given O_2 concentration (A_{cal}) is measured after equilibration of the reaction vessel by bubbling a gas calibration mixture containing a known oxygen concentration (for instance air, 20.8% O_2), and also after equilibration with a neutral gas (usually N_2 or Ar) to determine the m/z=32 background level (A_0) of O_2. When bubbling with the calibration gas, the reaction vessel should be slightly opened to the atmosphere to allow both pressure release and limited interaction of the atmosphere with the solution. Since the bubbling might last for more than 15-20 minutes, it should not be too strong to avoid evaporation or loss of solution via bubbles. The measurement of the calibration signals is done during the bubbling with sterile water or sterile buffered solution. A_{cal} and A_0 are obtained when the measured signal remains stable for minutes of bubbling. Real time concentrations (C(t)) of O_2 are then calculated as follows:

$$C(t) = (A(t) - A_0) \times \frac{C_{cal}}{(A_{cal} - A_0)} \qquad (12),$$

where t is time, A(t) is the amperometric signal and C_{cal} is the dissolved O_2 concentration in equilibrium with the calibration gas. C_{cal} can be calculated as:

$$C_{cal} = \frac{B_u \times C_{gas}}{V_m} \qquad (13),$$

where B_u is the Bunsen coefficient (volume of gas per volume of solvent), C_{gas} the volumetric proportion of O_2 in the gas and V_m is the molar volume of O_2. Values of B_u for different gases in solution in water at different temperatures are shown on **Table S1**. Note that if the calibration measurements are not done with water (e.g. salted water), the gas solubilities differ and other Bunsen coefficient apply. **Fig. S1** shows the signal for m/z=28, 32 during dark to light transition in *C. reinhardtii* and the corresponding concentrations of N_2 and O_2 inside the reaction vessel. Signal on the m/z=44 (N_2) is shown as a reference gas for further corrections.

Note here that in the case like NO where the gas is highly toxic, measurement of A_{cal} can be done by injecting a given volume of water (v) saturated with NO inside the closed reaction vessel already containing a volume of water free of NO (V). In this case, A_{cal} is measured as the peak signal measured and $C_{cal} = \frac{B_u \times C_{gas}}{V_m} \times \frac{v}{(v+V)}$.

In some cases, fragmentation tables of two gases overlap, which make it impossible to calculate gas concentrations from a single m/z. For example, the fragmentation table of N_2O overlaps those for N_2, NO and CO_2 (**Table 1**). In this case, CO_2 concentration is determined by using the m/z=12 signal, which represents 8.71% of the signal at m/z=44. The signal specific to N_2O at m/z=44 is calculated by subtracting the CO_2 component to the m/z=44:

$$A_{N_2O}(44) = A(44) - \frac{A_{CO_2}(12)}{0.0871} \qquad (14),$$

where $A_{N2O}(44)$ is the m/z=44 value due to N_2O, A(44) the total m/z=44 value and $A_{CO2}(12)$ the m/z=12 value. N_2O concentration can then be determined as described previously, using the $A_{N2O}(44)$ value for N_2O. Such calculations can be generalized to other gases showing overlapping fragmentation tables, although in some cases m/z values of more fragments are needed.

Table S1. Bunsen coefficient of various gases in water at various temperatures. Data shown are taken from the Gas Encyclopedia, L'air Liquide, 1976. More data can be found at https://encyclopedia.airliquide.com/

	0°C	*5°C*	*10°C*	*15°C*	*20°C*	*25°C*	*30°C*	*35°C*
H_2	0.0214	0.0203	0.0193	0.0185	0.0178	0.0171	0.0163	0.0158
N_2	0.02348	0.02091	0.01875	0.01705	0.01557	0.01441	0.01345	0.01264
O_2	0.0489	0.0429	0.0380	0.0342	0.0310	0.0283	0.0261	0.0246
NO	0.0774	0.0644	0.0571	0.05145	0.0470	0.0432	0.0410	0.0380
Ar	0.0286	0.0252	0.0224	0.0201	0.0183	0.0167	0.0154	0.0131
CO_2	1.7163	1.3735	1.1887	1.0106	0.8704	0.759	0.6678	0.5849
N_2O	ø	1.140	0.948	0.789	0.665	0.561	0.472	0.358

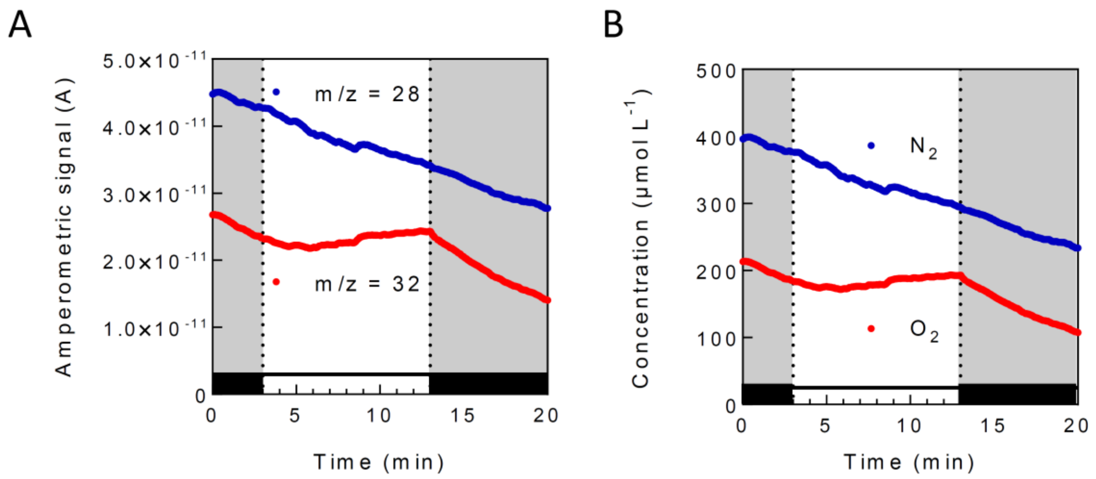

Figure S1 Amperometric signals for O_2 and N_2 measured with MIMS and calculated gas concentration during a dark to light transition in *Chlamydomonas* cells. A). Amperometric signal obtained for m/z = 28 and 32. **B**). N_2 and O_2 concentrations inside the reaction vessel. *C. reinhardtii* wild type cells were cultured as described in **Fig. 5**.

Methods S2: Determination of gas exchange rates

During MIMS measurements, the vacuum pump of the mass spectrometer continuously consumes gases contained in the reaction vessel, therefore the first derivative of gas concentration must be corrected by gas consumption to determine actual gas exchange rates occurring between the biological sample and the surrounding medium. At the membrane level, the limiting step of gas permeation is the diffusion (Konermann et al., 2008). The gas flow rate through the membrane (J) therefore follows Flick's law of diffusion:

$$J = D \times (C(t) - C_{vacuum}) \quad (15),$$

where D is a diffusion factor, C(t) the concentration inside the vessel and C_{vacuum} the gas concentration on the vacuum side of the membrane. Since C_{vacuum} is negligible compared to C(t), equation (15) leads to a simple first order differential equation in the absence of any other reaction occurring in the reaction vessel:

$$\frac{\partial(C(t))}{\partial t} = k \times C(t) \qquad (16),$$

where $\frac{\partial(C(t))}{\partial t}$ is the first derivative of C(t), and k the rate constant characterizing gas consumption, which is specific to each gas and each type of membrane. The rate constant k can be measured by injecting a known amount of the gas of interest in the closed reaction vessel. In the absence of other leak, the rate of decrease of the gas concentration is only due to the gas consumption by the mass spectrometer and C(t) follows equation (16) whose solution is:

$$C(t) = A + e^{kt} \text{ (17),}$$

one can rewrite equation (17) as:

$$\log\big(C(t)\big) = kt + a \quad (18)$$

Note that since the logarithm transforms a product into a sum, this slope can be calculated directly from the amperometric values:

$$\log\big(A(t)\big) = kt + a' \quad (19)$$

Practically, to measure A(t) one can either record the signal after closing the reaction vessel after bubbling water with a calibration gas like in **Methods S1**. Alternatively, in the case where the gas is toxic, like NO, this measurement can be done after injection of a given quantity of water saturated with NO inside the closed reaction vessel already containing a volume of water

free of NO, like in **Methods S1**. The signal A(t) can then be measured for 15-20 minutes to have enough data to fit it with linear regression.

After A(t) is recorded, log(A(t)) can be calculated and will therefore fit a linear curve (18). The slope of this linear curve gives k which is specific for each gas of interest. Note that k is a function of membrane properties but also the sample volume over the membrane surface; and needs therefore to be recalculated if the membrane or the sample volume is changed.

During experiments, variations of gas concentrations ($\frac{\partial(C(t))}{\partial t}$) in the reaction vessel result from both gas exchange by the biological sample and gas consumption by the mass spectrometer:

$$\frac{\partial(C(t))}{\partial t} = k \times C(t) + v(t) \qquad (20),$$

where v(t) is the gas exchange rate mediated by the biological sample, which can therefore be expressed as:

$$v(t) = \frac{\partial(C(t))}{\partial t} - k \times C(t) \qquad (21)$$

When experiments are carried out with diluted or weakly active samples, (diluted samples are preferred when measuring light-dependent reactions in order to limit the light heterogeneity in the sample), representing gas exchange by v(t) may be noisy (**Fig. S2**). In these conditions, a sliding average can be used to smooth the curve but leads to a decreased time resolution. Alternatively, corrected gas exchange can be expressed as cumulative gas exchange (cge(t)):

$$cge(t) = \int_0^t v(t)dt \quad (22)$$

cge(t) represents the concentration of gases that have been processed by the reaction of interest over all the reaction. However, it must not be confused with C(t) which is the actual concentration of gas present inside the reaction vessel. **Fig. S2** shows the gas exchange rate of N_2 and O_2 during dark to light transition in *C. reinhardtii* with and without a sliding average and the corresponding cumulated gas exchange.

Decreasing signal noise

In order to decrease signal noise that can result from fluctuations in the physical properties of different parts of the setup (membrane, stirring device, vacuum pump…), or fluctuations in temperature, the amperometric signal of an inert gas (usually nitrogen (N_2) or argon (Ar)) is recorded during the experiment and will be used as a reference. Ar is a good inert gas since it

cannot form any stable chemical compound in standard conditions (Khriachtchev et al., 2000). However, Ar concentration in the air is relatively low (0,93%) and can be consumed quickly at the membrane level. Therefore, N_2 is preferred as the reference gas for long experiments as far as it is not used by biological processes in the vessel. Assuming N_2 is inert, only consumption at the membrane is changing its concentration inside the vessel:

$$\frac{\partial(C_{N_2}(t))}{\partial t} = k_{N_2} \times C_{N_2}(t) \qquad (23)$$

During an experiment, some variations in the setup may slightly change the apparent gas consumption by the mass spectrometer, then, N_2 concentration follows the equation:

$$\frac{\partial(C_{N_2}(t))}{\partial t} = (k_{N_2} + \delta k_{N_2}) \times C_{N_2}(t) \quad (24),$$

where δk_{N2} is the variation of k_{N2} created by the setup fluctuation. Assuming all gases are subject to the same fluctuation in the same proportion, the gas exchange rate of the biological sample given in equation (21) is corrected by the N_2 signal as follows:

$$v(t) = \frac{\partial(C(t))}{\partial t} - k \times \frac{(k_{N_2} + \delta k_{N_2})}{k_{N_2}} \times C(t) \qquad (24)$$

Which can be rewritten as:

$$v(t) = \frac{\partial(C(t))}{\partial t} - k \times \frac{\frac{\partial(C_{N_2}(t))}{\partial t} \times \frac{1}{C_{N_2}(t)}}{k_{N_2}} \times C(t) \quad (25)$$

Equation (25) can be used based on any reference gas, as far as it is not reacting with the biological sample. **Fig. S2** shows the corrected and uncorrected cumulated gas exchange rate of N_2 and O_2 during dark to light transition in *C. reinhardtii*.

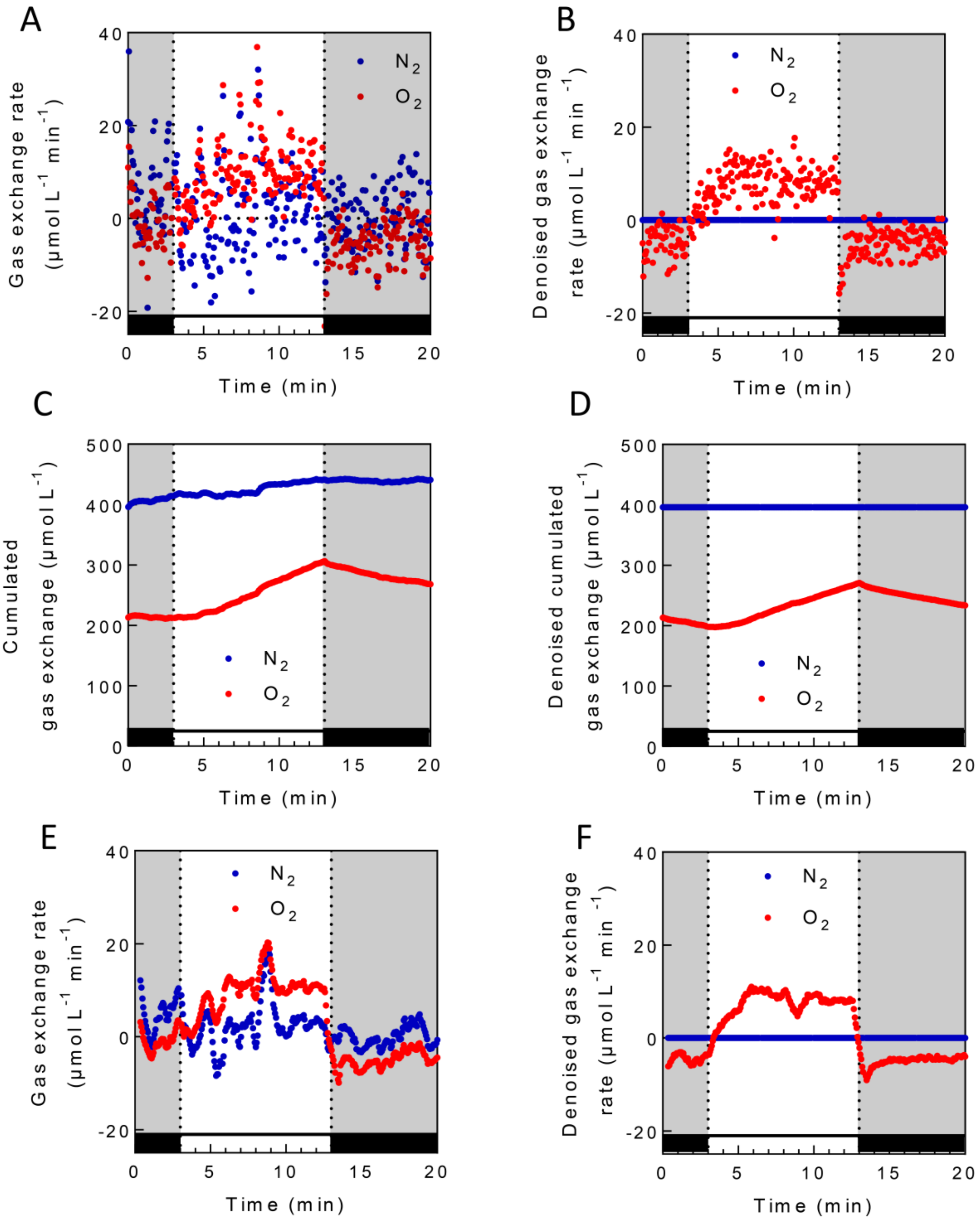

Figure S2. Determination of O_2 exchange from MIMS measurements upon correction from the mass spectrometer consumption (A, C and E) and subsequent correction using a reference gas (B, D and F). Gas concentrations obtained in Figure B1.1 are used to calculate O_2 exchange rates (**A, B**) and the corresponding cumulated O_2 exchange (**C, D**), additionally data from (**A** and **B**) where treated using a sliding average of 30s (**E, F**). Data were treated without (**A, C** and **E**) or with an additional normalization using N_2 as the reference gas (**B, D** and **F**).

References:

Khriachtchev L, Pettersson M, Runeberg N, Lundell J, Räsänen M (2000) A stable argon compound. Nature **406:** 874-876

Konermann L, Messinger J, Hillier W (2008) Mass spectrometry-based methods for studying kinetics and dynamics in biological systems. *In* TJ Aartsma, J Matysik, eds, Biophysical Techniques in Photosynthesis. Springer Netherlands, Dordrecht, pp 167-190
